# Methodological guide for assessing the carbon footprint of external beam radiotherapy: A single-center study with quantified mitigation strategies

**DOI:** 10.1016/j.ctro.2024.100768

**Published:** 2024-03-28

**Authors:** David Ali, Max Piffoux

**Affiliations:** a Centre de Radiothérapie et de Traitement des Tumeurs, Versailles, France; b Département d’Oncologie Médicale, Hospices Civils de Lyon, CITOHL, Lyon, France; c Direction de la Recherche Clinique et de l’Innovation, Centre Léon Bérard, Lyon, France

**Keywords:** External beam radiotherapy, EBRT, Radiotherapy, Radiation therapy, Irradiation, Carbon footprint, Carbon inventory, Environment, Greenhouse gas, Mitigation strategy, Mitigation strategies, Carbon accounting, Greenhouse gas accounting, Healthcare environmental impact

## Abstract

**Background and purposes:**

Data on the carbon footprint of external beam radiotherapy (EBRT) are scarce. Reliable and exhaustive data, including a detailed carbon inventory, are needed to determine effective mitigation strategies.

**Materials and methods:**

This study proposes a methodology for calculating the carbon footprint of EBRT and applies it to a single center. Mitigation strategies are derived from the carbon inventory, and their potential reductions are quantified whenever possible.

**Results:**

The average emission per treatment and fraction delivered was 489 kg CO₂eq and 27 kg CO₂eq, respectively. Patient transportation (43 %) and the construction and maintenance of linear accelerators (LINACs) and scanners (17 %) represented the most significant components. Electricity, the only energy source used, accounted for only 2 % of emissions.

Derived mitigation strategies include a data deletion policy (reducing emissions in 30 years by 12.5 %), geographical appropriateness (−12.2 %), transportation mode appropriateness (−9.3 %), hypofractionation (−5.9 %), decrease in manufacturers’ carbon footprint (−5.2 %), and an increase in machine durability (−3.5 %).

**Conclusion:**

Our findings indicate that a significant reduction in the carbon footprint of a radiotherapy unit can be achieved without compromising the quality of care.

This study provides a methodology and a starting point for comparison and proposes and quantifies mitigation strategies, paving the way for others to follow.

## Highlights

• The average emission per treatment and fraction delivered was 489 kg CO₂eq and 27 kg CO₂eq, respectively.
• Patient transportation (43 %) and the construction and maintenance of linear accelerators (LINACs) and scanners (17 %) represented the most significant components.
• Electricity, the only energy used, accounted for only 2 % of emissions.
• A significant reduction in the carbon footprint of a radiotherapy unit can be achieved without compromising the quality of care.

## Introduction

To understand how climate change might progress, several future scenarios with different degrees of optimism have been considered [1]. One such scenario from the Intergovernmental Panel on Climate Change (IPCC), SSP2-RCP4.5, is considered a plausible “middle of the road” scenario [2]. Under this scenario, global warming is projected to reach 2 °C around 2050 and 2.7 °C around 2090 [3]. The consequences of 2 °C of global warming are severe for both humans and ecosystems [3]. For example, 1.6 billion people will be exposed to heatwaves while being too economically vulnerable to adapt [4], 900 million people will experience severe water scarcity [5], and an additional 195 million people will be exposed to severe drought [6].

The consequences of 2.7 °C of global warming are expected to be more severe than those of 2 °C of global warming.

In OECD countries, the healthcare system is responsible for about 5 % of a nation's carbon footprint [7], [8], [9].

Determining how clinical practice decisions made by healthcare professionals impact the overall carbon footprint of the healthcare system is not an easy task. However, considering that the French healthcare system alone emits 46 Mt CO_2_eq, (about 8 % of the national emissions), it is clear that these decisions have a significant cumulative effect. Dividing this figure among the 226,000 French Medical Doctors (MD) results in an estimated carbon footprint of 203 t CO_2_eq per MD. Therefore, physicians and other healthcare professionals have the potential to significantly reduce the total carbon footprint by revisiting all procedures with a green perspective.

A detailed carbon inventory is required to define the most effective greenhouse gas reduction actions.

The carbon footprint of radiation therapy has already been evaluated in some studies [11], [12], [13], but the evaluated scope was narrow and the number of treatments considered was small.

The aim of this study was (a) to propose a methodology for calculating the carbon footprint of a radiotherapy unit with a broad scope, (b) to apply this methodology to calculate the carbon footprint of a single center (the Center of Radiotherapy of Versailles) and report results, and (c) to propose carbon mitigation interventions and estimate the carbon footprint reduction for each of them.

Full life cycle analysis is beyond the scope of this article. Only the carbon footprint will be discussed in this article, and only External Beam Radiotherapy (EBRT) will be discussed here. Brachytherapy is beyond the scope of this article.

## General methodology

### General framework

At a global level, we relied on the following resources: ISO 14064-2018 (the ISO standard for carbon accounting), the GHG Protocol [14] (an international multi-stakeholder partnership for carbon accounting), and the Bilan Carbone® methodology version 8 [15] (a French NGO for carbon accounting).

At the national (France) level, we mostly relied on the following resources: the Base Empreinte® database [16], the ADEME (Agence de l'Environnement et de la Maîtrise de l'Énergie) sectoral guide for the French healthcare sector [17], and the report from the Shift Project (a French NGO for carbon transitioning) on the French health sector [18].

These guidelines, which are hundreds of pages long and are meant to be applicable to all domains, are primarily designed for sustainability specialists. For radiation therapy workers to be able to apply these guidelines to the radiotherapy field, an understanding of three main concepts is necessary: functional perimeter (also known as functional boundary), organizational perimeter, and functional unit.

Below, we briefly describe these concepts and how they have been applied to our EBRT unit.

Defining the perimeter is essentially answering the question of what should be counted. According to ISO 14064, two types of perimeters should be defined: organizational and functional perimeters.

### Functional perimeter

Defining the functional perimeter is essentially answering the question of how far back one should go in the chain of carbon production. International standards usually break this down into three circles, called scopes:

• Scope 1: Direct emissions. For example, if a radiation unit has a gas furnace, the emissions from gas combustion will be included in Scope 1. Other direct emissions, such as sulfur hexafluoride (SF6), also fall into this scope.
• Scope 2: Indirect emissions for energy, cold, or heat production. Electricity, for example, is part of Scope 2 because if a center uses electricity that has been produced by a coal plant, the carbon emissions are produced in the plant but not locally in the center.
• Scope 3: This scope is sometimes divided into an Upstream scope and a Downstream scope.
• Upstream Scope 3: Basically, this scope includes every other emission that has been produced along the chain to finally provide the means to perform an activity. For example, if a sales employee from an accelerator company takes a plane to visit a center to sign a contract, emissions resulting from this activity will be included in Upstream Scope 3.
• Downstream Scope 3: This includes emissions that will be produced by others because of the activity. For example, emissions linked to the treatment of waste are part of Downstream Scope 3.

Scope 3 is by far the broadest, the most difficult, and the most important scope to capture and should not be excluded from the footprint.

In this study, we captured the broadest possible Upstream Scope 3 and some, but not all, aspects of Downstream Scope 3.

Care and drugs that are prescribed by the unit but delivered elsewhere are part of Downstream Scope 3. Because it would be very challenging to take into account the carbon footprint of healthcare that is delivered elsewhere and because this healthcare will be included in the footprint of the healthcare providers that deliver it, only care and drugs delivered inside the radiation unit have been included.

### Organizational perimeter

The organizational perimeter is the list of entities to be considered. For example, if a radiation therapy center is in a general hospital where some irradiated patients are hospitalized, should the hospitalization footprint be taken into account? Or if a patient has a follow-up visit in another hospital, should part of the footprint of that other hospital be considered?

#### Hospitalization

Some radiotherapy centers have their own hospitalization unit, while others outsource hospitalization. To ensure comparability between centers, we excluded hospitalization from the organizational perimeter.

#### Follow-up visits

Follow-up visit practices vary between centers. Some perform follow-up visits almost exclusively inside the radiation center, while others outsource most of these visits. To ensure comparability between centers, and because follow-up visits are not part of the treatment per se, we excluded them from the perimeter. Conversely, all other visits (initial consultation, dosimetry tomodensitometry (TDM), set-up visit, fraction delivery visits, and on-treatment visits) have been included.

### Functional unit

The functional unit is the basis to which all inputs and outputs of the system must refer. For example, for a yogurt factory, the functional unit would be one pot of yogurt. The total carbon footprint would be divided by the number of yogurts pots produced, and the emission per yogurt pot would be used to compare factories.

We propose to use one treatment course as the primary functional unit. However, because the treated disease sites and therefore the number of fractions per treatment course could be very different between centers, we also propose to use the delivered fraction as a secondary functional unit.

### Timeframe

Accounting was done for 12 months (from January 1, 2022, to December 31, 2022) to take seasonal activity and climatic changes into account.

### Emission categories

The GHG Protocol recommends reporting greenhouse gas emissions according to 23 categories. We initially reported the footprint following these guidelines (Supplementary Material Table 1). However, only a subset of these categories is relevant to EBRT.

Additionally, this classification did not provide a sufficient level of detail for determining mitigation strategies. Therefore, we proposed a new, more relevant, and specific radiotherapy classification. This classification consists of 19 categories: gas; oil; SF6; electricity; collective heating; medical consumables; desk consumables; drugs; servers; desk material; medical material; buildings and bunkers; IT material; waste; other purchased services; construction of Heating, Ventilation, and Air Conditioning (HVAC) systems; workers' transportation; patients' transportation; and construction of accelerators and scanners. The correspondence between the GHG Protocol and this radiotherapy classification, the emission factors, and the amortization periods used in this study are given in Supplementary Material Table 2.

### Methodology and results by category

#### Gas, oil, and collective heating

No gas, oil (gasoline or diesel), or collective heating was used in our unit in 2022.

#### Electricity

Electricity was the only direct and indirect source of energy. The monthly report of a specific electricity meter dedicated to the radiotherapy unit was used.

The total electricity consumption was 392,943 kWh. With an emission factor of 0.052 kg CO₂eq/kWh, the total emission for electricity (and therefore for the total direct energy consumption) was 20,433 kg CO₂eq (2.4 % of the unit’s total emission).

There was no sub-meter for the accelerators. The specification sheet of the manufacturers was used to determine the mean accelerator consumption by functioning hours with hypotheses on idle versus treatment time on a regular basis. The record and verify software was used to determine the total treatment time each month.

According to this methodology, the accelerators' electricity consumption accounted for 14 % to 28 % of the total electricity consumption and for 0.3 % to 0.6 % of the unit's emissions.

This last result is probably an overestimation because the manufacturers' specifications are prepared for installers to ensure sufficient power availability.

However, because we used a specific electricity meter dedicated to the radiotherapy unit, there are no substantial uncertainties about the total electricity consumption.

#### SF6

Sulfur hexafluoride (SF6) is a gas used in linear accelerators (LINACs). One kg of SF6 has a Global Warming Potential equivalent to 23,500 kg of CO_2_ [19].

SF6 contained in LINACs is mostly treated and recycled; however, leaks may occur. According to Chuter et al., [12] the average CO_2_eq emission due to one LINAC is 3,300 kg CO_2_eq/LINAC/year.

Our unit has three LINACs. SF6 leaks contributed to an emission of 9900 kg CO_2_eq (1.2 % of the unit’s emissions).

#### Accelerators and scanners

##### Construction and maintenance

We used public reports of the carbon footprint for Elekta (2022/23) [20] and Varian (2019) [21]. We excluded the part “use of sold products” because we believe that this can be better evaluated by the centers themselves. For each of these companies, a monetary ratio was calculated by dividing its carbon footprint by its total net sales. This methodology may be regarded as a type of environmentally extended input–output life cycle analysis.

These companies are dedicated to the radiotherapy (RT) equipment market, and their sales are mainly related to linear accelerators, scanners, or maintenance services. This estimator is therefore relatively unaffected by other kinds of revenues. Note that since RT facilities usually buy accelerators as well as maintenance contracts from these companies, the emission factor reflects both these services.

The result was remarkably similar for the two companies: 0.19 kg CO₂eq/dollar including maintenance. Monetary ratios are based on prices without VAT.

CO_2_ emissions associated with the construction of the machines occur during construction. To account for the machine's lifetime, these emissions are distributed over its lifespan (i.e., the amortization period). For accelerators and scanners, we used an amortization period of 12 years. Amortization periods used in this article are listed in Supplementary Material Table 2.

##### Use

Only machine construction and maintenance are considered here. The only energy used to supply the accelerators and scanners was electricity (as reported above).

##### Disposal

Because machine disposal was included in the contract, its footprint is already included in the monetary ratio.

The emission of the accelerators and scanners was 148,562 kg CO₂eq (17.3 % of the unit’s emissions).

This methodology does not constitute a full Life Cycle Analysis (LCA) of the machines used in our unit. However, currently, LCA of radiation therapy machines cannot be performed using publicly available data. In the meantime, the monetary ratio is considered a valid type of an environmentally extended input–output life cycle analysis proxy [22].

Additionally, it relies on the data released by the manufacturers and considers only the two main manufacturers. However, the remarkably similar results for both companies instill confidence in the monetary ratio results.

#### Server construction and maintenance

All our servers are physically located in our center but rented as a service.

The same methodology as for accelerators and scanners was applied. Public reports of Hewlett Packard's (the provider of our servers) carbon footprint for 2022 [23] were utilized, and a monetary ratio was calculated by dividing its carbon footprint by its total net sales. This methodology resulted in a value of 0.43 kg CO_2_eq/euro, which is in good agreement with the monetary ratio provided by ADEME. However, as with accelerators and scanners, we decided to exclude the emission category “product energy use,” leading to a monetary ratio of 0.28 kg CO_2_eq/euro. This emission factor encompasses the entire embedded carbon footprint of the servers, excluding their energy usage, which remains categorized under the electricity section.

The emission of the servers was 40,319 kg CO₂eq (4.7 % of the unit's emissions).

#### Buildings and bunkers

The carbon emission from the construction of healthcare buildings is 1,147 kg CO₂eq/m^2^ [10]. The total radiotherapy area, including bunkers, has been considered.

Bunkers contain a large amount of concrete, whose production has a significant carbon footprint. To account for this, bunker construction plans were used to determine concrete volume. The emission factor for concrete is 155 kg CO₂eq/ton [16]. The volumetric mass of concrete used in bunkers typically ranges from 2 to 4 tons per cubic meter. We used a mean value of 3 tons. An amortization period of 30 years was used.

The emission of buildings and bunkers was 53,122 kg CO₂eq (6.2 % of the unit's emissions).

#### Patient transportation

Our unit has no transportation services that could potentially collect multiple patients in one journey.

Patient transportation mode was not registered in 2022. However, a sample of 179 patients treated at the beginning of 2023 was recorded. The transportation modes were individual car (n = 155; 87 %), ambulance (n = 15; 8 %), public transportation (n = 8; 4 %), and walking (n = 1; 0.6 %).

Because patients using ambulances do not always come from their homes but sometimes from a hospitalization unit, the files of these patients were manually checked to determine the exact origin of these patients. For other patients, their home addresses were used.

For each of these patients, the distance to the unit (one way) was calculated using the Google Distance Matrix API [24]. Since transportation mode was not independent of distance and the number of fractions (one-way ANOVA), the total distance and total ride emissions for these 179 patients were calculated taking into account their transportation mode and the emission factors listed in Supplementary Material Table 2. In the sample, the mean emission factor was 0.2152 kg CO₂eq/km.

Then, for each treatment in 2022 (1753), the Google Distance Matrix API was used to retrieve the GPS coordinates of the patient’s home address. The results were cross-validated with the use of the Laposte API [25]. The Google Distance Matrix API was then used to determine the distance by road between the GPS coordinates of the patient’s home address and our radiotherapy unit.

Distances of more than 100 km were excluded from the analysis because we believe that these patients do not come from their permanent residences but are temporarily residing in accommodations closer to the unit during their treatment.

The distance could be determined for 1701 treatments. The mean distance was 22 km (n = 1701). For the remaining 52 treatments, a mean distance of 22 km was used. The total distance for each patient was the number of visits (initial consultation, dosimetry TDM, set-up, and fractions) multiplied by the above-calculated distance. An emission factor of 0.2152 kg CO₂eq/km as determined above was used.

The total emission of patients' transportation was 364,700 kg CO₂eq (42.5 % of the unit’s emissions).

This methodology has four main limitations. First, the use of a sample in 2023 to determine the transportation mode of patients in 2022 raises concerns about the representativeness of the data. However, we believe that there is no reason for the distribution of transportation modes to change significantly between 2022 and 2023.

Second, the home addresses of the patients were used to calculate distances, but patients may also travel from their workplaces. However, even though this was not prospectively recorded, we believe that a very small percentage of our patients were working during their treatment. Moreover, the effect of replacing home addresses with work addresses would likely average out.

Third, the distances traveled by empty taxis to pick up patients at their homes or at the radiotherapy center were not considered. Only the distances between the home address and the radiotherapy unit were considered. This leads to an underestimation of the actual emissions of taxis.

Lastly, even though carpooling is theoretically possible, and this was not prospectively recorded, we believe that the number of patients who used this transportation mode is negligible.

#### IT, Medical, and desk material

To ensure exhaustiveness, two approaches were used for IT, medical, and desk material: every item physically present in the unit was counted, and the balance sheet of assets was checked. The emission factors listed in Supplementary Material Table 2 were used.

Emissions were as follows: for medical material, 54,037 kg CO₂eq (6.3 % of the unit's emissions); for IT material (except servers), 16,637 kg CO₂eq (1.9 % of the unit's emissions); and for desk material, 2,267 CO₂eq (0.2 % of the unit's emissions).

#### HVAC construction

Radiation departments often need heavy climatization installations. We used a monetary ratio of 0.7 kg CO₂eq/euro (the ADEME monetary ratio for heavy machinery) and an estimated lifetime of 7 years as the amortization period.

Only HVAC construction is considered here. The only energy used to supply the HVAC system was electricity (as reported above).

The emission of HVAC construction was 47,756 kg CO₂eq (5.6 % of the unit's emissions).

This result is highly sensitive to the monetary ratio chosen. Since we did not find any validated monetary ratio specific to HVAC construction, we used the generic monetary ratio for heavy machinery provided by ADEME. However, because the parts account for only 5 % of the total unit's emissions, we believe that the choice of this monetary ratio has a small impact on the calculation of the total emissions.

#### Medical consumables

The medical consumables purchased were retrieved from the data sheets of the purchasing department. We used the emission factors listed in Supplementary Material Table 2.

For drugs, a monetary ratio of 0.5 kg CO₂eq/euro has been used [17]. Because it is technically very difficult to keep track of all prescribed drugs, prescribed drugs have been excluded from the scope, and only on-site delivered drugs have been considered.

The emission of medical consumables was 21,861 kg CO₂eq (2.5 % of the unit's emissions).

#### Other purchased services

Two other purchased services have been considered: laundry service and software licenses.

For laundry, we used an emission factor of 0.37 kg CO₂eq/kg [18].

For software licenses, we used a monetary ratio of 0.4 kg CO₂eq/euro [26]. For time-limited licenses (subscriptions), we used the license period as the amortization period. For perpetual licenses, an amortization period of 5 years was used.

The emission of other purchased services was 15,946 kg CO₂eq (1.9 % of the unit's emissions).

#### Workers’ transportation

A survey was sent to all workers asking for their address, number of working days, and transportation mode (including training, education, continuing professional development, congress visits, etc.). The response rate was 48 %. Distances were calculated using Google Maps, and the same emission factors as for patients were used. The results were extrapolated to non-responding workers.

The emission of workers’ transportation was 41,066 kg CO₂eq (4.8 % of the unit's emissions).

A non-response bias cannot be entirely ruled out: there is a possibility that employees more concerned about environmental issues are more likely to respond to the survey and may also be more likely to choose low-carbon transportation options. This bias would likely lead to an underestimation of the true carbon footprint. However, the workers who responded most frequently to the survey were physicians (71 % of physicians responded), and they are the workers who travel the most for work.

#### Waste

Our center had no high-risk waste in 2022. We used an emission factor of 0.367 kg CO₂eq/kg [16] for household wastes.

Dumpster volume was considered to have a density of 0.2 kg/l. Garbage disposal was 3 times a week.

The total emission of waste was 15,115 kg CO₂eq (1.8 % of the unit's emissions).

#### Total carbon footprint

##### Activity – functional unit

In 2022, the unit delivered 1,753 treatments and 31,860 fractions. Patients made 36,994 visits to the unit for initial consultation, dosimetry TDM, set-up, and fractions.

##### Total emissions

The total emission was 857,476 kg CO₂eq. The average emission per treatment was 489 kg CO₂eq, the equivalent of a one-way flight from Paris to New York [27]. The average emission per fraction was 27 kg CO₂eq.

**Fig. 1:**
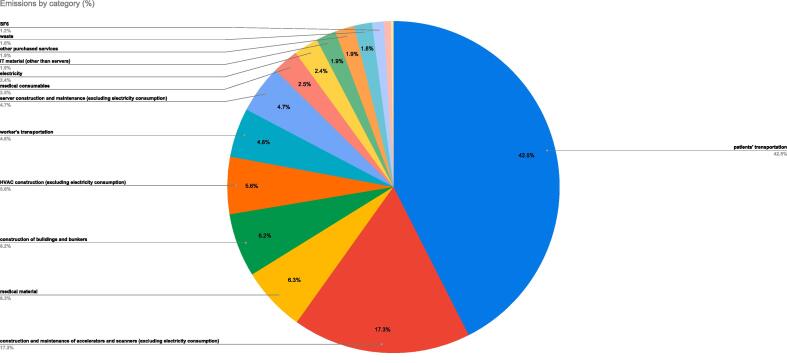
Emissions by category.

Emissions by category are shown in Table 1 and Fig. 1.

**Table 1:** Emissions by category.

**Category** **(radiotherapy classification)**	**Emissions** **(% of total)**	**Emissions** **(kgCO2Eq by year)**
patients' transportation	43 %	364,722
construction and maintenance of accelerators and scanners (excluding electricity consumption)	17 %	148,562
medical material	6 %	54,037
construction of buildings and bunkers	6 %	53,122
HVAC construction (excluding electricity consumption)	6 %	47,756
workers' transportation	5 %	41,066
server construction and maintenance (excluding electricity consumption)	5 %	40,319
medical consumables	3 %	21,861
electricity	2 %	20,433
IT material (other than servers)	2 %	16,637
other purchased services	2 %	15,946
waste	2 %	15,115
SF6	1 %	9900
desk consumables	1 %	5734
desk material	0 %	2267
oil	0 %	0
gas	0 %	0
drugs	0 %	0
collective heating	0 %	0
Total		857,476

#### Mitigation strategies

##### Carbon footprint of machine manufacturers

Scanner and treatment machine construction accounted for 17.3 % of the unit’s total carbon footprint. Manufacturers could adopt mitigation strategies to lower their own carbon footprint. For example, a 30 % reduction in their carbon footprint would lead to a 5.2 % reduction in the unit’s total emissions.

##### Machine durability

Improving the durability of machines would also lower their footprint. Here, we used a 12-year amortization period for accelerators. With a 15-year amortization period, the total carbon footprint of the center would be reduced by 3.5 %.

##### Evaluation of the impact/benefit ratio of new machines

The construction of more complex treatment machines would result in larger greenhouse gas emissions. For example, to the best of our knowledge, the only clinical benefit proven by a randomized study of LINAC MRI so far is a reduction in acute toxicity for prostate treatment [28]. The clinical benefits of these machines should be thoroughly evaluated and carefully weighed against their environmental impact before widespread use in all radiotherapy units across the globe.

##### Hypofractionation

A classical treatment comprises an initial consultation, a dosimetry TDM, a set-up, and 1 to 40 fractions. Patients' transportation emissions are linearly linked to the number of fractions. Moreover, if hypofractionation leads to a sufficient reduction in delivered fractions, the number of accelerators needed for a given amount of treatment could be decreased, lowering machine construction emissions.

We used the following hypotheses:

• Adjuvant breast radiotherapy:
  o No nodal RT, age >75 years: 5 fractions [29]
  o Nodal RT or age <75 years: 15 fractions [30], [31] (a boost, if indicated, would be a Simultaneous Integrated Boost) [32], [33], [34], [35]
• Prostate: 20 fractions [36], [37]
• Prostatic bed: no change
• Bone metastasis: 1 fraction [38], [39]
• Stereotactic Body Radiation Therapy (SBRT): no change
• Other treatments: no change

Compared with using the actual treatment performed in our unit, using this treatment scheme strictly would decrease the number of fractions to 27,507 (−14 %), which is not enough to eliminate one machine. Carbon emission savings would only be obtained by a reduction in patients’ transportation. Patients' transportation emissions would decrease to 324,531 kg CO₂eq, a reduction of −5.9 %.

Conversely, with no hypofractionation at all were used, i.e. under the following hypotheses:

• Adjuvant breast radiotherapy: 25 fractions ± boost in 8 fractions if indicated
• Prostate: 39 fractions
• Bone metastasis: 10 fractions
• Stereotactic Body Radiation Therapy (SBRT): no change
• Other treatments: no change

The total number of fractions would increase to 36,866. Patients' transportation emissions would rise to 403,880 kg CO₂eq. Assuming that this rise would not necessitate the acquisition of a new accelerator, the increase in emissions would be +3.4 %.

##### Geographical appropriateness

Patients are not always treated in the radiotherapy unit closest to where they live. Geographical appropriateness, i.e., treating patients in the unit closest to where they live, could lead to a significant reduction in patient transportation distance. For each treatment, we calculated the distance between the patient's origin and the five closest radiotherapy units. Our center was the closest one for about 55 % of the treatments. For the remaining treatments, if patients were systematically treated in the closest radiotherapy unit and assuming that this unit had the same carbon footprint as ours, the carbon footprint of their treatment would be reduced by 12.2 %.

##### Appropriateness of transportation mode

For each treatment, we calculated the transportation time with public transportation. If each patient using an individual car, with a difference in transportation time between car drive and public transportation of less than 15 min, had taken public transportation, the total emissions would have been reduced by 9.3 %.

##### Combined mitigation strategies

If Hypofractionation, Geographical Appropriateness and Appropriateness of Transportation Mode were combined, patients' transportation emissions would decrease to 188,660 CO₂eq, a reduction of 21.7 %.

##### Data deletion policy

Servers’ emissions represent 4.7 % of the total emissions. This is mainly due to the large amount of data stored for long periods of time.

Currently, the average amount of data stored is 3 GB per treatment. For security reasons, our unit has two backups. With a compression or deduplication rate of 30 %, each treatment leads to a data storage of 7 GB. Our current total data storage size is 67,662 GB (including backups).

In France, for adults, the law stipulates that medical data need to be stored for 20 years after the last visit if the patient is still alive, or 10 years after the death date if the patient is deceased [40].

We made a model of the amount of data stored in our unit given three different data storage policies:

1. No data deletion (our current data storage policy)
2. A simple data deletion policy: erase data 20 years after the last visit
3. An advanced data deletion policy: erase data based on three parameters: the date of the last visit, the date of death (if deceased), and the type of data (detailed rules are given in Supplementary Material Table 3).

The follow-up function was historically retrieved from our database.

The death date was retrieved from the INSEE database [41] for 12,402 patients treated in our institution with the use of the DCD system [42] with a score cut-off that leads to a specificity of >99 % and a sensitivity of 80 %.

The follow-up function, survival function, and data deletion policy were used to simulate the size evolution of our data storage under the three data deletion policies.

We hypothesized that the number of treatments per year, the data storage per treatment, and the emissions by 1 GB of stored data would be constant over time.

In 30 years, the servers’ emissions would represent 19 % of the unit’s emissions without any deletion policy, 12 % with the simple deletion policy, and 7 % with the advanced policy.

The amount of data stored, the corresponding emissions (if emissions/GB are stable), and the proportion of the unit's total emissions (if other emissions are stable) currently and in 30 years under the three data deletion policies are summarized in Table 2.

**Table 2:** Data deletion policy effect.

	**Data size** **(Gb)**	**Server emissions** **(kg CO2eq)**	**Proportion of the** **unit's emissions**
Current	67,662	40,319	5 %
in 30 years: no deletion policy	320,094	190,739	19 %
in 30 years: simple deletion policy	183,058	109,081	12 %
in 30 years: advanced deletion policy	108,362	64,571	7 %

##### Care appropriateness

The definition of appropriate healthcare is complex [43], [44], [45], [46]. Basically, it may be summarized as “the right care, to the right person, at the right time” [47].

According to an old survey, French physicians consider about 28 % of care to be “not fully justified” [48]. At the patient level, overuse of healthcare is considered inappropriate since the patient is exposed to risks and side effects that do not exceed the benefits. At the society level, according to the OECD, about 20 % of health expenditure makes little or no contribution to improving people's health [49].

Healthcare overuse is detrimental to patients, society, and the environment.

Proposals for indicators of healthcare overuse have already been made in several other medical specialties. For example, in medical oncology, the proportion of patients receiving chemotherapy in their last 14 days of life or starting a new chemotherapy regimen in the last 30 days has been considered an indicator of overly aggressive care [50], [51], [52]. However, such indicators are lacking in radiotherapy.

Defining and registering indicators of radiotherapy overuse may help to improve the quality of care by providing physicians’ feedback [53].

##### Communication with patients, healthcare workers, and industry

Most of the mitigation levers are in the hands of physicians: selection of new machines or techniques, hypofractionation, geographical appropriateness, definition of a data deletion policy, care appropriateness, and machine durability.

Manufacturers, industrial producers, and IT providers also have an important role to play by lowering their own carbon footprint, improving machine durability, and providing the means to implement the data deletion policy defined by the physicians.

Public authorities also play a role by defining health data conservation rules and encouraging appropriate care through control, regulation, guidelines, or price policies. Additionally, they can define the care access map to minimize transportation needs and improve transportation mode appropriateness through reimbursement policies.

Each stakeholder group may have a role in selecting the new machines to be purchased from manufacturers.

Patients also play an important role in geographical and transportation mode appropriateness.

Mitigation strategies require actions and efforts from all these actors. Communication on this topic with all the actors involved is essential for the efforts to be accepted.

##### Other mitigation strategies

Other mitigation strategies were found to have a negligible impact, such as suppression of SF6 leaks (−1.2 %), reducing by half the LINAC electricity consumption (−0.4 %), and replacing all light bulbs with LED (−0.1 %).

While SF6 leaks appear to be a significant problem when considered in isolation [54], their role in the overall carbon footprint of EBRT diminishes when other emission sources are taken into account. Although any feasible reduction is valuable, focusing on low-impact measures could potentially detract from pursuing more impactful mitigation strategies such as those previously discussed.

**Fig. 2:**
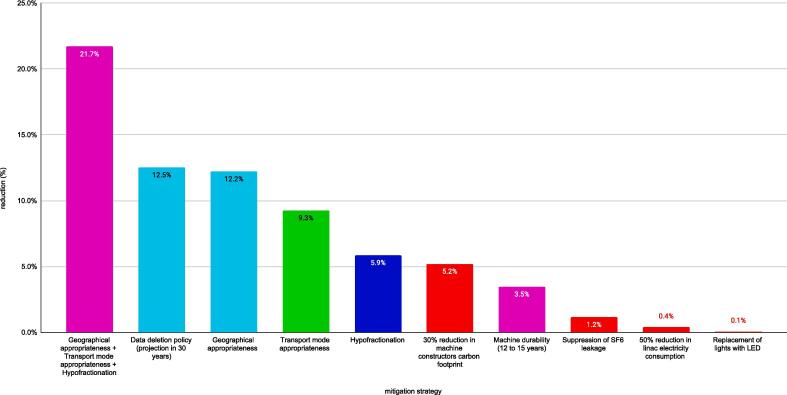
Quantified mitigation strategies.

Table 3 shows the proposed mitigation strategies and their quantification if evaluated, and Fig. 2 shows the impacts of quantified mitigation strategies.

**Table 3:** Mitigation strategies.

Mitigation strategy	Reduction (%)	Actor	Note
Communication with patients, workers, and industrial partners	n.a.	all	necessary for the efforts the be accepted
Care appropriateness	n.a.	physicians	indicators to be defined
Geographical appropriateness + Transportation mode appropriateness + Hypofractionation	21.7 %	physicians/patients	
Data deletion policy (30-year projection)	12.5 %	physicians/manufacturer	30-year projection
Geographical appropriateness	12.2 %	physicians/patients	treatment in the closest center
Transportation mode appropriateness	9.3 %	patients	public transportation if individual car was used and travel time difference is at most 15 min
Hypofractionation	5.9 %	physicians	see text for hypothesis
30 % reduction in machine constructors' carbon footprint	5.2 %	manufacturer	assuming a 30 % reduction in companies' carbon footprint
Machine durability (12 to 15 years)	3.5 %	physician/manufacturer	transitioning from 12 to 15 years machines lifespan
Suppression of SF6 leakage	1.2 %	manufacturer	Source: Chuter, 2023
50 % reduction in LINACs' electricity consumption	0.4 %	manufacturer	assuming that Linear accelerator (LINAC) electricity consumption is one-third of total electricity
Replacement of lights with LED	0.1 %		assuming LED lighting has negligible consumption (as it's 10 times less than halogen)

## Discussion

Emission factors are country specific. Therefore, the main caveat of this study is that its findings cannot be applied to other countries. However, the general methodology may be valid worldwide provided that the emission factors are adapted.

Some aspects of Downstream Scope 3 have been excluded, such as prescribed drugs and imaging, as well as follow-up visits.

The methodology described here is mostly valid for external beam radiotherapy and may not apply to brachytherapy.

Also, this is not a full life cycle assessment (LCA). Only the carbon footprint has been considered.

Our unit is a stand-alone facility whose only activity is EBRT. Therefore, it provides a good opportunity to specifically determine the carbon footprint of EBRT.

The carbon footprint of radiation therapy has already been evaluated in some studies, but the scopes considered were more narrow, leaving out what has been shown in our study to have a major impact. Moreover, these previous studies were limited to a few dozen patients, while our study included all treatments delivered over a full year, totaling over 1750 treatments.

Based on our current understanding, the most comprehensive study on this topic is the one conducted by Chuter et al., which examined five emission sources (electricity, patient travel, some medical consumables, and SF6 leakage). However, our study expanded this scope to encompass 19 potential emission sources. The Chuter et al. study did not include several key sources with notable impacts, including the construction and maintenance of LINACs and servers, buildings and bunkers, and HVAC constructions. As a result, a direct comparison of the two studies is not possible.

Therefore, the Chuter et al. study cannot be employed as a methodological guide for carbon accounting in radiotherapy units or as a baseline for comparison. In contrast, we believe that our study can be used for these purposes.

To the best of our knowledge, this is the most comprehensive report on EBRT carbon footprint, either in terms of scope or in terms of the number of treatments. It provides a methodology and a baseline for comparison and proposes 9 mitigation strategies. The potential reductions were quantified for 6 of them.

Our findings indicate that a significant reduction in the carbon footprint of a radiotherapy unit can be achieved without compromising the quality of care.

We hope that this study will pave the way for others to calculate their own carbon footprint using our methodology, derive their most efficient mitigation strategies, and/or apply the mitigation levers proposed here.

A public Google Sheet is available to help others calculate their carbon footprint:

https://docs.google.com/spreadsheets/d/1lfSq6K1Dabbkt7TPkTNsMbUpFrrRkbG1Sl8g_mX5eLk/edit?usp=sharing

## Funding support

No dedicated funding.

## Patient consent statement

This study did not utilize any clinical or medical data. Instead, it relied on statistically analyzed, anonymized group data. Under French law, this approach falls outside the scope requiring specific patient consent forms.

## CRediT authorship contribution statement

**David Ali:** Conceptualization, Methodology, Software, Validation, Formal analysis, Investigation, Resources, Data curation, Writing – original draft, Writing – review & editing, Visualization. **Max Piffoux:** Conceptualization, Methodology, Writing – review & editing.

## Declaration of competing interest

The authors declare the following financial interests/personal relationships which may be considered as potential competing interests: David Ali: founder of Risoft and conceptor of the DCD system. Max Piffoux: has shares and scientific advisor for Everzom, Evora bioscience, Therafast-Bio.
